# The Evaluation of Biomarkers of Physical Activity on Stress Resistance and Wellness

**DOI:** 10.1007/s10484-022-09538-2

**Published:** 2022-03-16

**Authors:** Arpine Muradyan, Tanja Macheiner, Marine Mardiyan, Eduard Sekoyan, Karine Sargsyan

**Affiliations:** 1 Department of Physical Rehabilitation, Armenian State Institute of Physical Culture and Sport, Aleq Manukyanstreet 11, Yerevan, Armenia; 2 International Biobanking and Education, Medical University of Graz, Elisabethstraße 38/Merangasse 11, 8010 Graz, Austria; 3 Department of Public Health and Health Organization, Yerevan State Medical University, Koryun Street 2, Yerevan, Armenia; 4 Department of Rehabilitation, Physiotherapy and Sports Medicine, Yerevan State Medical University, Koryun Street 2, Yerevan, Armenia; 5 Department of Medical Genetics, Yerevan State Medical University, Koryun Street 2, Yerevan, Armenia; 6 National Medical Research Radiological Centre of the Ministry of Health of the Russian Federation, Moscow, Russia

**Keywords:** Autonomic nervous system, Physical fitness, Stress management, Well-being

## Abstract

Physical activity can improve health as well as reduce stress and the risk of developing several widespread diseases. However, there exists no accepted standard biomedical examination-method for stress evaluation. The purpose of this study was to investigate the effect of regular physical activity on stress and wellness as well as the evaluation of potential biomarkers in this field. This study included 105 people (mean age = 36.57 ± 1.4 years) who were randomly assigned into the exercise group 1 (EG-1) (n = 41), the exercise group 2 (EG-2) (n = 30), and the control group (CG) (n = 34). Measurements of stress and wellness were obtained by Multiscan BC-OXI before and after experimental period. This device presents a multifrequency segmental body composition 3D analyser with digital pulse oximeter. The key indicators of stress as well as for wellness were significantly improved in the EG-1. Parasympathetic activity showed significant changes as potential stress biomarker. Statistically significant gender differences were not observed in the comparable groups. The results suggest that the stress resistance and well-being significantly improved in the EG-1 due to regular physical activity. However, further research is necessary to determine effects of physical activity on integral health indicators.

**Supplementary Information:**

The online version contains supplementary material available at 10.1007/s10484-022-09538-2.

## Introduction

The evolutionary perspective on physical activity (PA), fitness and health states that human anatomy and physiology have remained relatively unchanged over the past 40,000 years. In general there is a wide recognition of the relevance of PA in health promotion and quality of life (Loureiro and Veloso, 2017) which is substantiated by existing research such as the positive effects of regular PA on body and psyche through production of the “Brain derived neurotrophic factor” (BDNF) (Noakes & Speeding, 2012; Stults-Kolehmainen & Sinha, 2013). According to the World Health Organization (WHO) stress and low PA are two of the leading contributors for a premature death in developed nations (World Health Organization, 2018, 2020). Additionally, intensity of PA is inversely and linearly associated with mortality (Lee & Skerrett, 2001). Furthermore, men and women who reported increased levels of PA and fitness, showed reductions of 20% to 35% in relative risk of death (Macera et al., 2003; Macera & Powell, 2001). In the Behavioural Risk Factor Surveillance System (BRFSS) database, the number of unhealthy days reported by 175,850 adults was inversely associated with PA. Therefore, those who exercise, suffer less from depression, anxiety, fatigue and cognitive impairments (Stults-Kolehmainen & Sinha, 2014). The most studied of all stress related disorders is cardiovascular disease (CVD), which has been highlighted as the leading cause of mortality worldwide (World Health Organization, 2011), including a graded linear relation between the volume of PA and health status, so that the most physically active individuals have the lowest risk (Warburton et al., 2006). Other previous studies even show that exercise can relieve stress, depression and improve cognitive function (Noakes & Speeding, 2012; American Psychological Association, 2020; Hamer, 2012).

Further previous studies, combining multiple PA assessments are recommended (Haskell, 2012; Melanson & Freedson, 1996), including PA as adjuvant in psychotherapy for increase of wellbeing and reduction of symptoms (Aylett et al., 2018; Karg et al., 2020; Wegner et al., 2020).

Wellness is a term that encompasses an individual’s outlook on life and well-being, including their perceptions of personal PA, happiness, learning, society, work and spirituality (Corbin et al., 2013).The WHO defined wellness as the optimal state of health of individuals and groups (Smith et al., 2006). Wellness is a dynamic integration of somatic and mental well-being, grounded in the expanding awareness. Within this perspective, the amalgamations of these lifestyle components play a role in our total well-being, including the management of stress (Ratliff Dawson, 2004). For the evaluation of stress, heart rate variability (HRV) analysis is commonly used as a quantitative marker depicting the activity of the autonomic nervous system (ANS) related to mental stress (Tonello et al., 2014). Moreover, physical or mental imbalance caused by harmful stimuli can induce stress to maintain homeostasis. During chronic stress, the sympathetic nervous system is hyperactivated causing physical, psychological and behavioral abnormalities. However, previous research detected the HRV as a potential biomarker for psychological stress indicator (Kim et al., 2018; Tonello et al., 2014), as well as for psychological and physiological resilience and health (Young & Benton, 2018).

Pulse waveform, arterial stiffness and cardiac output can be measured by the oximeter, which is based on photoelectric plethysmography. Previous studies showed the potential of these parameters as stress biomarkers in the past (Lahiri et al., 2008; Vasu et al., 2015).The Galvanic Skin Response (GSR) is defined as a change in the electrical properties of the skin and corresponds to electrical conductivity of the skin and is used for the estimation of the sudomotor innervation, which is the cholinergic innervation of the sympathetic nervous system in sweat glands (Joshi & Kiran, 2020; Nepal et al., 2018). Therefore the GSR measurement can be considered to be a simple and useful tool for examination of the autonomous nervous system function, and especially the peripheral sympathetic system.

Bioelectrical impedance analysis (BIA) is a relatively simple, inexpensive and non-invasive technique to measure body composition and is therefore suitable in field studies and larger surveys (Böhm & Heitmann, 2013). Under alternating electrical excitation, biological tissues produce a complex electrical impedance which depends on tissue composition, structures, health status and applied signal frequency and therefore bioelectrical impedance methods can be utilized for non-invasive tissue characterization. The impedance analysis conducted over a wide frequency band provides more information about the tissue interiors which support us to better understand the biological tissues anatomy, physiology and pathology (Bera, 2014). Multiscan BC-OXI is a new, innovative type in medical measurement and analysis systems to investigate bioelectrical impedance, galvanic skin response, pulse waves and heart rhythm. Therefore, the Multiscan BS-OXi includes various features such as Segmental Body composition 3D Analyzer, body composition assessment (e.g. Body Mass Index), microcirculation assessment, body composition 3D Modeling (fat mass colour code), Diet adviser including visceral fat 3D Modelling, Vertebral 3D Modeling, Extended assistance to the therapeutic decision for Fitness/Sport trainings, HRV indicators, Stress/Fatigue assessment and digital pulse wave analysis. The Multiscan BC-Oxi stress index (correspond to stress resistance) includes a wide range of physiological stress parameters (see Table 1). It is a non-invasive test that takes only a few minutes.

**Table 1:** List of multiscan BC-Oxi stress index indicators

Stress indicators	Normal range
Heart Rate	Standard indicator for age and gender
SDNN^a^	≥ 40 ms
RMSSD^b^	35–65 ms
RR intervals	Standard indicator for age and gender
Stress index	≤ 200.0 Conv. Un
pNN50	10.0–49.0%
*Valsalva test*	> 1.18
K30/15	> 1.10
Blood pressure response	< − 10 mm Hg
Total power	> 780.0 ms^2^
Power low frequency	22.0–46.0%
Power high frequency	22.0–34.0%
LF/HF	≤ 2.0 Conv. Un

^a^standard deviation of all normal-to-normal R-R intervals

^b^Root mean square of successive differences

At present, a wide range of theoretical approaches exists but no accepted standard biomedical examination method for stress evaluation. Therefore, this study aimed to find a reproducible, standardized measuring method for physical stress and wellness assessment as well as to investigate the effect of PA on stress and wellness by using the Multiscan to evaluate potential stress biomarker. For this reason, the PA protocol, based on previous research (Taylor, 2013; US Department of Health and Human Services, 2018) include (i) a warm up training (20 min), cross training in middle level (20 min), strength training (20 min) and swim training as additional option one time per week. According to the Multiscan BC-OXi method, this is the first study that is conducted to analyze the results of stress resistance and wellness.

Considering that the Multichan Multiscan BC-OXi is often used in fitness and health institutions in different countries we decided to conduct this triple blind study to analyze and evaluate by this broad used device.

## Methods

### Subjects and Study Design

A triple-blind, randomized, controlled trial was conducted with 105 participants aged 16–71 years. The average age of the study population was 36.57 ± 1.4 years and 54.3% were female participants.

A sample size of 30 subjects was calculated to detect a change. An *α*-value of 0.05 and *β*-1 of 0.8 were assumed as well as a 10% dropout rate. Based on the literature data on practice, we initially hypothesized that regular physical activity classes would contribute to improving stress tolerance and wellness indicators. For this purpose, we selected 41 participants according to the method. The change was detected by a comparative analysis of the results of the study before and after the inclusion of clients in the physical activity program.

The inclusion criteria for participation in the study were healthy condition, absence of medication, and consent of the participants. In the case of minors, the agreement was signed by their parents.

The exclusion criteria included diagnosed diseases during decompensation, cardiovascular and pulmonary insufficiency, contraindications to PA or currently used medications.

Before the experiment started, all participants signed a subscription agreement to visit the fitness club, which specifies all the rules, precautions, services and a fitness testing (bioimpedance research by the Multiscan BS_OXI device). In the fitness and wellness club where the study was carried out (Multi Wellness Center), there are several turnstiles from the entrance to the exit that can only be opened with a wristband. Each club visitor has his own wristband. With the program implemented in customer service, we can see when, where and how long this customer stayed at the club, as well as how many times per week he went to one-on-one training with a trainer. Group workout attendance data was obtained from group workout trainers. With the help of medical control and fitness testing, customer service, management and marketing departments, as well as trainers and clients were selected for the first, second and third groups. Data on attendance at group training sessions were taken from the group trainers.

After a preliminary study of all participants, they were divided into three groups: EG-1 involved 41 participants with a mean age of 37.8 ± 2.2 years, EG-2 involved 30 participants with a mean age of 35.2 ± 2.7 years and CG with a mean age of 36.3 ± 2.2 years. EG-1 group (n = 41) included supporters of PA of two to four times a week, EG-2 group (n = 30)—individuals who engaged a fitness program once a week or less frequently, and the CG included individuals without any fitness programme at all.

Comparable age and gender groups were ensured, so that age-related differences had no effect on the level of change in the variables studied and all groups are homogeneous by gender. Details about age and gender distribution of participants in groups are shown in Table 2.

**Table 2:** Distribution of participants in comparable groups by age and gender

Comparable groups	*n*	Male		Female	
		*n*	M ± m	*n*	M ± m
EG-1	41	20	38.3 ± 3.1	21	37.6 ± 3.3
EG-2	30	15	34.5 ± 4.3	15	35.9 ± 3,4
CG	34	13	34.7 ± 4.1	21	37.2 ± 2,6
Total	105	48	36.0 ± 2.1	57	37.0 ± 1.8

### Physical Activity

The participants of EG-1 and EG-2 were divided into three subgroups for training. The "Global Recommendations on PA for Health" according to the WHO address three age groups: 5–17 years old (*n* = 4, 3.8%), 18–64 years old (*n* = 97, 92.4%) and 65 years old and above (*n* = 4, 3.8%). These age groups were selected, while taking into consideration the nature and availability of the scientific evidence relevant to the prevention of non-communicable diseases through PA (Barwais et al., 2013).

The combined fitness training programme, the frequency of fitness activities (60 to 90 min a day, from two to four times a week) and the duration (from 3 to 12 months) were selected individually for each participant based on age, level of PA and the functional state of the body by experts (World Health Organization, 2010).

The CG subjects did not participate in any intervention and maintained their PA preliminary levels throughout the study. All participants in this group had low physical activity, which according to the criteria of the Multiscan BC-OXi program is registered as low PA-office activity.

Additionally to the recommendations of the doctor and the trainer, the client chooses the fitness programs that he wants and prefers to go to. Naturally, the fitness club has different programs for different age groups and we believe it is inappropriate to force the client to go to workouts he does not want to attend. Therefore, the choice of training is up to the client. The duration, intensity and load is recommended by the doctor and trainer, and at the same time, the age, gender and physical fitness of the client must be taken into account.

All the clients passed the fitness test on the Multiscan BC-Oxi both before and after 3 to 12 months after starting the training. Since the clients had different season tickets (3 months, 6 months, annual), they had their physical activity/fitness programs for different durations and went for the repeated fitness test at different times. This information is easily verified, since all information about the date of the tests is stored in the customer database. From about 5,000 club visitors, we selected only those whose data were strictly consistent for the sample of three separate groups.

In the specified fitness club, each new client must pass a medical control and fitness testing before starting with classes in the club, with mandatory recommendations for fitness classes [duration, frequency (60 to 90 min/day, 2–4 days a week), intensity, etc.] based on age, gender and physical fitness. Group training coaches also teach classes simultaneously for three categories of clients—beginners, intermediate and advanced (during the workout the trainer gives clear instructions for the three categories marked). Individual training sessions also take the clients’ age and level of physical fitness into account (individual approach).

Furthermore, the examination procedure was carried out uninterruptedly in four stages. The first three stages (baseline recording, Valsalva manoeuvre and deep breathing) were carried out in a sitting position and the fourth stage (orthostatic test) in a standing position. Stage 1 (baseline recording—2 min) includes that the probands sit, do not move or speak, and are relaxed. Stage 2 (Valsalva manoeuvre—45 s) means that the probands take a deep breath, hold the air for 15 s, then exhale and try to relax. Stage 3 (deep breathing 1 min) contains that the probands slowly inhale and exhale air every 5 s and stage 4 (orthostatic test—50 s) that the probands stands up and put their finger into the Pulse Oximeter.

### Physiological Stress and Wellness Measurement

Multiscan BC-OXI is a portable multi frequency segmental Body composition 3D analyser with Digital Pulse Oximeter and was used for detection of stress and wellness biomarker based on ACSM’s Guidelines for Exercise Testing and Prescription (Pescatello et al., 2014).

The intended use relates the role of healthy lifestyle with helping to reduce the risk or impact of certain chronic diseases or conditions. Such information was considered to optimize a wellness and SPA treatment plan and correct its intensity. By using the Multiscan BC-OXI, a broad panel of biomarker was detected. These included an estimation of body composition by bioimpedance, RR intervals based on the HRV feature in the spectral analysis, provided by mathematical analysis of the input of the sinus node depolarization as an estimation of the autonomic nervous system activity, pulse waveform, which is provided by the oximeter, based on photoelectric plethysmography (including estimation of arterial stiffness and cardiac output), analysis of the galvanic skin response as estimation of sudomotoric responses to the post sympathetic cholinergic system electrical stimulation, analysing of oxygen saturation (SPo2 feature) and HR (Heart Rate). For the evaluation, a record of cardio heart rate intervals, obtained from a heart rate sensor, is used. The application measures stress through the activity growth of the sympathetic component of the autonomic nervous system. The algorithm is based on the analysis of the frequency domain of HRV. Therefore, indicators of the stress score were: HR Valsalva ratio (a lower value is an indicator of decreased baroreceptor activity), K30/15 (Indicator of orthostatic hypotension and vasovagal syndrome), HF% (main indicator of parasympathetic activity) and LF/HF (balance of activity of sympathetic/parasympathetic NS). The higher the stress score, the better the indicators or some of the indicators that summarize this score. This means that the body resistance to stress is high. We interpreted the grading indicators based on the results of the Multiscan method for assessing stress, the normal range of indicators is 70–100, the critical limits are 35–70, and the violation is 0–30.

Among other parameters Multiscan BC–OXI evaluates the HRV Indicators and stress management (see Supplemental material 1) and Wellness score. The ANS scores are shared in “ANS a Score” (a stands for activity) and “ANS b Score” (b stands for balance). The severity of the ANS activity and balance are quantitated using an ANS score-algorithm for fast interpretation of results. The ANS/HRVa score includes (i) standard deviation of all normal-to-normal R-R intervals (SDNN) (Normal range: 40 to 80 ms) (ii) Root Mean Square of Successive Differences (RMSSD) reflects the variations in heart rate and the stability of the heart rhythm (normal range: 35–65 ms) and (iii) Total Power (TP) shows the overall autonomic activity.

The stress indicators of the ANS/HRV b score include (i) Low Frequency (LF) as strong indicator for sympathetic activity, (ii) High Frequency (HF) as indicator for parasympathetic activity and (iii) the LF/HF ratio (normal range 0.5 to 2%). K30/15 presents an indicator of vaso-vagal syncope as part of the parasympathetic test (normal range: > 1.10%).

The GSR is defined as a change in the electrical properties of the skin. The stress score (normal range = 70–100) and wellness score (normal range = 80–100) by the Multiscan BC-OXI analyser are rated on a scale from 100 (excellent) to 0 (bad) accordingly the device description by the manufacturer. Segmental body composition analysing is part of the wellness score, including fat (free) mass, lean muscle mass, intra- and extracellular water, basal metabolic rate and body mass index (BMI).

The measurement by Multiscan BC-OXi was carried out before the start of the fitness programs, and repeated testing was carried out no earlier than three months (as noted above, it depended on the type of season ticket). Since the season ticket includes two free tests for 6- or 12-month subscriptions, customers themselves decided when to pass the test. Repeated testing of each proband was carried out at approximately the same hour as the first one (e.g. if the first test was in the morning, then the second one was also carried out in the morning). The study under the Multiscan BC-OXi program lasts from two to six minutes and during this time all parameters, including HRV, are measured. During the measurement, the client goes through four stages, the first three sitting and the last stage standing. The data were received immediately after the end of the program.

### Statistical Analysis

Statistical data analysis was carried out using primer of the biostatistics version 4.03 by Stanton A. Glantz. All data are presented as mean ± standard deviation. Normality of data distribution was assessed using the student’s t-test and the Wilcoxon signed-rank test. According to the Wilcoxon criterion for indicators of stress management, the group size was sufficient to apply the normal distribution in EG-1 (W = 818, Z = 5.507; *p* = 0.000), in EG-2 (W = 6, Z = 0.070; *p* = 0.944) and CG (W = 115, Z = 1.305; *p* = 0.192).

According to the Wilcoxon criterion for indicators, the wellness score was sufficient in EG-1 (W = 421, Z = 3.182; *p* = 0.001), EG-2 (W = 180.0, Z = 1.945; *p* = 0.052) and CG (W = 189.0, Z = 1.943; *p* = 0.052).

As a result of applying the W coefficient, we obtained a normal distribution, which gave us the right to use student's t-test to assess the reliability of the results. The reliability of the results was estimated with a probability of 95% or higher (*p* < 0.05). The reliability of the results was evaluated by the student's t-test.

For investigating the relationship between the correlation variables, we used Pearson's correlation analysis method.

## Results

This study included 105 people from 16 to 71 years who were randomly assigned into EG-1 (n = 41), EG-2 (n = 30), and CG (n = 34) participants accordingly the sample size calculation and physical activity level.

According to the results of PA intervention in the framework of the combined fitness training program, the initial indicators of stress score in EG-1 group were 54.4 ± 1.2 (σ = 7.6), in EG-2 group were 65.2 ± 1.5(σ = 8.0) and in CG were 61.6 ± 1.46(σ = 8.7).

After the stress score experiment, the stress scores were increased in EG-1 (69.4 ± 1.1; σ = 7.0) (see Supplemental material 1) and CG (63.4 ± 1.5; σ = 9.0) groups. In EG-2 the stress score was reduced (64.7 ± 1.6; σ = 8.6) after experimental period (see Table 3).

**Table 3:** Stress score (normal range = 70–100) measured at baseline and after study in comparison groups

Stress score	EG-1 (*n* = 41)		EG-2 (*n* = 30)		CG (*n* = 34)	
	Baseline	After	Baseline	After	Baseline	After
M ± m	54.4 ± 1.2	69.4 ± 1.1	65.2 ± 1.5	64.7 ± 1.6	61.5 ± 1.46	63.4 ± 1.5
σ	7.6	7.0	8.0	8.6	8.7	9.0
	T = 9.3, *p* = 0.000 Z = 5.507, *p* = 0.000		T = 0.233, *p* = 0.816 Z = 0.070, *p* = 0.944		t = 0.885, *p* = 0.379 Z = 1.305, *p* = 0.192	

Based on the results of the Student's t-test, the significant differences were detected in EG-1 (*p* = 0.000). In EG-2 and CG no statistically significant differences were found (p = 0.816 and *p* = 0.379 respectively).

A comparative analysis of stress score indicators by gender in all three groups before and after the experiment was also conducted (see Table 4).

**Table 4:** Stress score analysis before and after study for men and women

Stress score	Male					
	EG-1 (*n* = 20)		EG-2 (*n* = 15)		CG (*n* = 13)	
	Baseline	After	Baseline	After	Baseline	After
M ± m	54.1 ± 2	68.7 ± 1.6	64.9 ± 2.4	62.5 ± 2.6	58.4 ± 2.3	63.2 ± 2.8
σ	8.8	7.1	9.4	10	8.3	10.3
	t = 5.8, *p* = 0.000		t = 0.7, *p* = 0.504		t = 1.3, *p* = 0.202	
Stress score	Female					
	EG-1 (*n* = 21)		EG-2 (*n* = 15)		CG (*n* = 21)	
	Baseline	After	Baseline	After	Baseline	After
M ± m	54.71 ± 1.4	70.05 ± 1.5	65.5 ± 1.7	66.9 ± 1.6	63.4 ± 1.9	63.6 ± 1.8
σ	6.5	7	6.5	6.3	8.6	8.4
	t = 7.4, *p* = 0.000		t = 0.599, *p* = 0.554		t = 0.076, *p* = 0.94	

The general pattern of the study by gender has been preserved. Based on the results of the student's t-test for stress score the results were as follows: Between EG-1 and EG-2, the indicators were reliable (*t* = 2.504, *p* = 0.015); between EG-1 and CG were also reliable (*t* = 3.291, *p* = 0.002), between EG-1 and CG were unreliable (*t* = 0.593, *p* = 0.555).

These results prove that significant and reliable changes in the stress score were only in EG-1. Furthermore, after experimental phase EG-1 showed statistically significant differences to the CG in LF/HF (*t* = 1.96, *p* = 0.03), SDNN (*t* = 2.29, *p* = 0.01) and TP *(t* = − 2.89, *p* < 0.01). Moreover, the differences of these parameters in EG-1 before and after were statistically significant again (*p* < 0.01). Supplemental material 1 demonstrates the stress score results of one participant of EG 1.

According to the results of the wellness score experiment, the initial indicators of stress score in EG-1 group were 81.78 ± 0.7 (σ = 4.6), in EG-2 group were 83.47 ± 1 (σ = 5.5) and in CG were 84.9 ± 0.9 (σ = 5.2).

After the stress score experiment, the wellness scores in EG-1 and CG groups were increased [EG-1 84.29 ± 0.7 (σ = 4.9) and CG 85.17 ± 0.7 (σ = 4.6)]. In EG-2 the score was reduced (82.53 ± 1 (σ = 6.1) (see Table 5).

**Table 5:** Wellness score (normal range = 80–100) measured at baseline and after study in comparison groups

Wellness score	EG-1 (*n* = 41)		EG-2 (*n* = 30)		CG (*n* = 34)	
	Baseline	After	Baseline	After	Baseline	After
M ± m	81.78 ± 0.7	84.29 ± 0.7	83.47 ± 1	85.17 ± 0.8	84.09 ± 0.9	82.53 ± 1
σ	4.6	4.8	5.5	4.6	5.2	6.0
	t = 2.535, *p* = 0.013 Z = 3.182; *p* = 0.001		t = 1.327, *p* = 0.190 Z = 1.945; *p* = 0.052		t = 1.160, *p* = 0.250 Z = 1.943; *p* = 0.052	

In the CG, the stress and wellness score differences were insignificant.

Based on the results of the student's t-test, the data in EG-1 were reliable (*p* = 0.013). Statistically significant differences were detected in EG-1(*p* = 0.013). Differences in EG-2 and CG were statistically insignificant (*p* = 0.190 and *p* = 0.250 respectively). Again, reliable data were obtained indicating that the wellness score and the stress score were improved only in EG-1.

If the sample size increases, the average stress coefficient (M) would fluctuate between 82.89 and 85.69.

In the wellness score research, we conducted a comparative analysis of gender indicators in all three groups before and after the experiment. The wellness score showed statistically significant increases between measurements before and after the experimental period in EG-1 for males (n = 20; *t* = 2 and *p* = 0.05) and females (n = 21; *t* = 2 and *p* = 0.05).

Statistically significant differences in wellness score coefficient were observed in the EG-1 male and female group. In CG and EG-2 no statistically significant differences in the wellness score coefficient were found.

In the next stage of the study, the correlation between changes in the stress score and the integral indicator of the health wellness score was investigated.

We applied the Pearson correlation analysis method. There was no reliable correlation between the trends of changes in the studied parameters. The correlation coefficient was in EG-1 Rxy = 0.2, *p* = 0.2, in EG-2 Rxy = 0.165, *p* = 0.382 and CG Rxy = 0.125, *p* = 0.483.

The coefficient of combined correlation in groups EG-1 and EG-2 was Rxy = 0.149, *p* = 0.2; in groups EG-1 and CG was Rxy = 0.2, *p* = 0.08.

## Discussion

The purpose of this study was to evaluate the relationship between PA, stress management and wellness score with the help of the Multiscan BC-OXI.

PA and fitness programmes can improve health stress resistance, reduce the risk of developing several diseases, reduce stress, depression and anxiety. Most importantly, regular activity can improve the quality of life. Higher levels of PA are associated with better Health Related Quality of Life (HRQoL). Using an objective measure of PA compared to the subjective one, shows a relatively better HRQoL (White et al., 2009). An increase in physical fitness will reduce the risk of premature death, and a decrease in physical fitness will increase the risk (Erikssen, 2001) from cardiovascular disease in particular among asymptomatic men and women. Furthermore, a dose– response relation appears to exist, so that people with the highest levels of PA and fitness have the lowest risk of premature death (Warburton et al., 2006). A physically active population is important for the health of both the individual and society, with sport participation being one, increasingly important, motivator for exercise (Malm et al., 2019).

Data of previous studies identified PA as a variable associated with higher well-being (Rodriguez-Fernández et al., 2016; Kipp, 2016; Kleszczewska et al., 2018).

Although this association was not found in the case of negative effect when frequency of PA, the results indicate that in order to achieve high levels of psychological well-being, individuals should engage in PA on a regular basis, at least three to four times a week. Health effects of PA in many cases follow a dose–response relationship; dose of PA is in proportion to the effect on health (Hills et al., 2015; Kujala et al., 1998).

Furthermore, many studies have confirmed the benefits of any type of physical exercise or sport for reducing negative emotion (Landers & Petruzzello, 1994; McAuley et al., 1996; Anokye et al., 2012; Lawton et al., 2017).

Social wellness makes individuals able to have successful interaction, communication, relationship and to feel appreciated and belonging (Kramer, 2015).

The results of our research showed that regular PA, increased the normative indicators of the stress score and the wellness score (arterial assessment, ANS assessment, lifestyle assessment) especially in EG-1 (Table 5); including an increased parasympathetic activity due to a decreased LF/HF ratio. Moreover an increased SDNN value (the EG-1 mean value increased from 40 to 50 ms) represents short-term variability and is associated with decreased mortality since sympathetic and parasympathetic activities contribute to SDNN (Shaffer & Ginsberg, 2017).

Based on the analysis of the results of our and other researchers, we hypothesise that probably a low risk of premature death and higher body resistance to stress in individuals with high levels of PA may be associated with an improvement in their indicators of stress and wellness scores.

To the best of our knowledge, this is the first study detailing the effect of exercise (fitness program) on stress indicators and wellness parameters and detailed description of the relationship between indicators of stress and wellness scores as a result of using fitness programs according to the WHO PA recommendations for specific age groups.

In our study, we included gender distribution in all three groups and by results no significant gender differences were observed in the comparable groups. This means that there are no gender differences in assessing stress and wellness in PA.

The significance of the research results in EG-1, participants who strictly followed the above recommendations, allows to apply the method for a wider selection.

Significant changes in stress and wellness indicators in EG-1 proves that it is the regularity of fitness programmes that is an important criterion for improving indicators, reducing which leads to stress and increase the indicators of health. Reliable data was received, indicating that the stress score indicator improved only in EG-1within the normal range. With participants in EG-2, who engaged in fitness once a week or less, the indicators of the stress score were decreased, and the wellness score increased. However, in both cases the differences were insignificant. If the sample size increases, the average stress coefficient (M) would fluctuate between 67.2 and 71.6.

The findings on the correlation between changes in the stress score and the integral indicator of the health wellness score are regardless of the principles of group formation: In other words, there is no reliable correlation between changes in the average stress index and the average level of the integral indicator that assesses health status. It cannot be concluded that trends in improving the average stress score lead to an improvement in the average score of an integral indicator that assesses health status. Since the integral indicator that evaluates health state consists of several components, a reliable correlation between some components of the integral indicator and the average changes in the wellness score is possible. We will try to substantiate this hypothesis in our further research. Furthermore the results indicate for further research on the relationship between stress, physical activity and wellness practices (i) to employ different research methodologies to investigate physical activities influences on wellness practices and stress levels, (ii) Control group experimental designs should be considered the effects of physical activities on stress levels and the relationship between stress and wellness practices and (iii) to follow WHO "Global Recommendations on PA for Health", including three age groups to improve indicators for stress and wellness indicators. In conclusion, our results suggest that regular PA is associated with improvement of body stress resistance and wellness. Future research is recommended to identify the impact of physical activity on integral health indicators for novel application. We consider the possibility of using this Multiscan technique to determine the state of physiological stress and wellness (the totality of physiological indicators) as a non-invasive, user-friendly and fast innovative method.

Limitations of the study include the fact that the Multiscan BC-Oxi uses proprietary algorithms to reflect the stress and wellness score. However, this can lead partly to unclear interpretations of results about individual biomarker such as parasympathetic and sympathetic marker. For instance the Vasalva maneuver can reflect the baroreflex function but cannot provide an index of baroreceptor function without the use of a beat-to-beat blood pressure monitoring. In addition, a detailed evaluation of potential stress biomarker is difficult without normative database.

Moreover an increase of PA by focusing on endurance training (e.g. swimming, walking, dancing, and cycling) (Grässler et al., 2021) seems to be necessary for a better analysing of PA effects on stress resistance.

## Supplementary Information

Below is the link to the electronic supplementary material.

Supplementary file1 (DOCX 35 kb)

## Data Availability

All data are stored in a database without external access by the first author.
