# Supplementary material for: The Evaluation of Biomarkers of Physical Activity on Stress Resistance and Wellness
Source: Appl Psychophysiol Biofeedback. 2022 Mar 16;47(2):121–9. doi: 10.1007/s10484-022-09538-2 (PMC9098580; doi:10.1007/s10484-022-09538-2)
Supplement: Supplementary file 1 — Supplementary file1 (DOCX 35 kb) [file 10484_2022_9538_MOESM1_ESM.docx]

Supplemental table

|  |  |  | **INDICATORS OF STRESS SCORE** | | | | | | | | | | | | | | | | Stress score N=70-100 | |
| --- | --- | --- | --- | --- | --- | --- | --- | --- | --- | --- | --- | --- | --- | --- | --- | --- | --- | --- | --- | --- |
| **EG -1 Pat** | **Gender** | **Age** | **HR   60-90 bpm** | | **SDNN   >=40ms** | | **RMSSD   35-65ms** | | **RR intervals  667-1000m** | | **Stress Index <=200.0 Conv. Un.** | | **pNN50   10.0-49.0%** | | **Тotal power >780.0 msec2** | | **LF/HF  <=2.0 Conv. Un.** | |  |  |
|  |  |  | Bef | Aft | Bef | Aft | Bef | Aft | Bef | Aft | Bef | Aft | Bef | Aft | Bef | Aft | Bef | Aft | Before | After |
| **1** | f | 40 | 74,1 | 77,4 | 63,7 | 60,3 | 51,5 | 58,6 | 810,1 | 775,6 | 62,6 | 69,2 | 31,5 | 40,7 | 2581,5 | 2831,4 | 1,1 | 1,1 | 69 | 75 |
| **2** | m | 26 | 84,8 | 71,5 | 38,6 | 45,2 | 51,8 | 64,1 | 707,8 | 839,5 | 160,9 | 102,2 | 31,9 | 47,8 | 608 | 913 | 2,1 | 0,7 | 52 | 75 |
| **3** | m | 42 | 77,4 | 63 | 43,8 | 58,9 | 56,9 | 68,7 | 775 | 952,6 | 6,8 | 59,6 | 38,5 | 53,8 | 922,6 | 2131,5 | 2,1 | 0,9 | 43 | 56 |
| **4** | f | 31 | 96,2 | 83,4 | 28,6 | 47,6 | 37,7 | 47,1 | 623,5 | 719,1 | 285,7 | 116,1 | 13,5 | 25,8 | 332,4 | 1593,6 | 1,5 | 2,1 | 56 | 63 |
| **5** | m | 27 | 93,2 | 79,9 | 46 | 42,4 | 44,4 | 47,3 | 644,1 | 750,6 | 174,5 | 133,9 | 22,2 | 26 | 1760,5 | 1158,3 | 1 | 0,9 | 57 | 68 |
| **6** | m | 33 | 92,5 | 77,3 | 46,1 | 46,6 | 42,7 | 61,1 | 649 | 776,5 | 156,6 | 99,3 | 20,1 | 43,9 | 1487,7 | 1228,1 | 2,1 | 2,1 | 45 | 63 |
| **7** | m | 33 | 71,5 | 61,8 | 59,2 | 81,7 | 56,9 | 66,7 | 839 | 971,2 | 69,4 | 30,8 | 38,4 | 51,2 | 2012,8 | 4792,7 | 1,1 | 0,4 | 66 | 72 |
| **8** | m | 35 | 88,2 | 74,3 | 31,8 | 64,3 | 45,9 | 61,7 | 680,1 | 807,8 | 254 | 54,1 | 24,1 | 44,7 | 394,8 | 2990,3 | 2,3 | 1 | 52 | 75 |
| **9** | f | 30 | 101,8 | 80 | 35,8 | 64,9 | 32,5 | 61,5 | 589,4 | 809,1 | 188,1 | 54,2 | 6,8 | 44,6 | 793,4 | 2992 | 2,2 | 0,9 | 52 | 75 |
| **10** | f | 28 | 86,9 | 80,7 | 41,7 | 54 | 50,9 | 65,7 | 690,4 | 743,8 | 159,3 | 93,7 | 30,6 | 49,9 | 918,6 | 2040 | 0,8 | 0,9 | 63 | 75 |
| **11** | m | 31 | 76,6 | 65,3 | 62,6 | 76,5 | 56,9 | 65 | 783,8 | 918,7 | 81,4 | 48,8 | 38,4 | 49 | 3092,6 | 4403,2 | 2,1 | 0,6 | 49 | 75 |
| **12** | m | 20 | 90,7 | 77,4 | 33,5 | 48,7 | 39,6 | 56,4 | 661,8 | 774,8 | 280,9 | 112,7 | 16 | 37,8 | 682,9 | 1480,5 | 2,2 | 2,1 | 52 | 63 |
| **13** | f | 24 | 91,1 | 89,6 | 36,8 | 48,6 | 46,3 | 53,5 | 658,3 | 669,5 | 185,3 | 123,2 | 24,7 | 34,1 | 670,2 | 1509,8 | 1,5 | 0,9 | 62 | 70 |
| **14** | f | 54 | 79,9 | 91,3 | 34,6 | 27 | 39,5 | 34,3 | 750,9 | 656,8 | 158 | 185 | 15,9 | 9,1 | 736,2 | 362,5 | 2,2 | 0,7 | 49 | 57 |
| **15** | f | 27 | 74,1 | 63,7 | 62,7 | 43,6 | 51,5 | 56,2 | 810,1 | 941,6 | 62,6 | 105,5 | 31,5 | 37,6 | 2581,2 | 1193,3 | 1,1 | 0,8 | 68 | 75 |
| **16** | f | 69 | 74,8 | 73,3 | 28,3 | 43,5 | 38,1 | 57,3 | 802,5 | 818,1 | 219,7 | 128,4 | 14,1 | 39 | 323,5 | 1031 | 1,5 | 0,8 | 63 | 75 |
| **17** | m | 70 | 76,1 | 61,7 | 32,3 | 73 | 46 | 93,7 | 788,2 | 972,9 | 191 | 43 | 24,3 | 86,3 | 488,1 | 3294,7 | 2,1 | 0,9 | 52 | 75 |
| **18** | m | 40 | 73,2 | 67,7 | 39 | 61,7 | 35,4 | 84,5 | 819,8 | 886,3 | 131,4 | 61,4 | 10,5 | 74,4 | 1088,9 | 2238 | 2 | 0,8 | 52 | 75 |
| **19** | m | 63 | 54,9 | 39,8 | 78,1 | 84,1 | 100 | 95,7 | 1093,5 | 1509,3 | 48,3 | 38,2 | 95,1 | 88,9 | 3910,1 | 4105,5 | 0,6 | 0,3 | 70 | 75 |
| **20** | m | 38 | 87,7 | 81,3 | 32,8 | 49,3 | 35,6 | 49,5 | 684,2 | 737,7 | 242,5 | 94,6 | 10,7 | 28,9 | 1702,6 | 1702,6 | 1,5 | 2,1 | 54 | 63 |
| **21** | f | 24 | 106,9 | 101,4 | 31,8 | 42,4 | 33,2 | 36,5 | 561,3 | 591,9 | 255,8 | 177,5 | 7,6 | 11,9 | 717,3 | 1386,2 | 2,3 | 1 | 52 | 75 |
| **22** | f | 59 | 111,4 | 98,4 | 28,9 | 26,8 | 40,8 | 31 | 538,8 | 610,1 | 342,1 | 219,4 | 17,6 | 4,8 | 282,7 | 442,6 | 2,4 | 2,5 | 52 | 52 |
| **23** | f | 50 | 101,6 | 103,4 | 25,9 | 38,4 | 31,8 | 30,7 | 590,3 | 580,5 | 387,8 | 194,8 | 5,9 | 4,4 | 261,5 | 1061,1 | 1,5 | 1 | 55 | 63 |
| **24** | f | 49 | 85,4 | 76,2 | 34,9 | 45,7 | 44,9 | 44,6 | 202,2 | 787,2 | 188,8 | 120,3 | 22,8 | 22,4 | 658,4 | 1475,8 | 2,2 | 2,1 | 52 | 63 |
| **25** | m | 30 | 97,1 | 89,1 | 38,7 | 44,4 | 36,7 | 41,4 | 618,2 | 673,4 | 166 | 143,9 | 12,3 | 18,3 | 1096,3 | 1634,6 | 2,1 | 1 | 40 | 65 |
| **26** | m | 34 | 88,1 | 77,7 | 49 | 56 | 41,5 | 50,8 | 680,8 | 772,2 | 113,9 | 95 | 18,4 | 30,5 | 1891,8 | 2438,8 | 1,1 | 1,1 | 64 | 75 |
| **27** | f | 26 | 93,3 | 77,7 | 48,8 | 56 | 42,1 | 50,8 | 642,9 | 772,7 | 147,4 | 95 | 19,2 | 30,5 | 1737,8 | 2438,8 | 1,1 | 1,1 | 52 | 74 |
| **28** | f | 69 | 74,9 | 69,6 | 34,2 | 50,7 | 38,8 | 57,5 | 801,2 | 861,8 | 183,7 | 81,7 | 15 | 3,2 | 654,9 | 1813,6 | 2,2 | 0,9 | 52 | 75 |
| **29** | f | 36 | 97,3 | 82,9 | 28,2 | 41,9 | 33,8 | 41,9 | 616,5 | 723,7 | 286,2 | 146,8 | 8,4 | 19 | 316,4 | 1119,1 | 1,5 | 0,9 | 50 | 69 |
| **30** | f | 34 | 92,1 | 86 | 37,5 | 45,9 | 35 | 45,5 | 651,7 | 697,6 | 179,6 | 137,4 | 10 | 23,7 | 1028,2 | 1429 | 2,1 | 1 | 50 | 75 |
| **31** | m | 59 | 94,2 | 75,2 | 27,5 | 45,3 | 35,4 | 42,5 | 636,9 | 797,7 | 277,1 | 116,6 | 10,6 | 19,7 | 388,1 | 1504,3 | 2,5 | 1 | 52 | 72 |
| **32** | m | 30 | 84,5 | 79,2 | 49,4 | 51 | 44,6 | 41,9 | 710,3 | 757,8 | 99,6 | 88,9 | 22,4 | 18,9 | 1742,2 | 1998,7 | 1,1 | 1,2 | 64 | 75 |
| **33** | f | 22 | 95,1 | 71,4 | 36,2 | 53,7 | 34,2 | 58 | 631 | 840,2 | 18,3 | 81,2 | 9 | 39,9 | 767,5 | 2060,7 | 1,5 | 1 | 44 | 62 |
| **34** | f | 19 | 117 | 80,3 | 26,8 | 44,5 | 29,1 | 59 | 512,8 | 747,3 | 310,4 | 127 | 2,1 | 41,2 | 350 | 1066,9 | 1,5 | 0,8 | 52 | 73 |
| **35** | f | 43 | 92,1 | 84,9 | 37,5 | 42,7 | 39,1 | 33,6 | 651,6 | 707 | 235 | 115,1 | 15,3 | 8,2 | 1007,7 | 1310,2 | 2,1 | 1,5 | 52 | 75 |
| **36** | f | 29 | 99,9 | 93,8 | 37,9 | 41,3 | 48,4 | 52,3 | 600,7 | 639,8 | 187,7 | 151,9 | 27,4 | 32,5 | 608,7 | 876,6 | 1,5 | 0,9 | 52 | 75 |
| **37** | m | 26 | 93,7 | 86,5 | 40,9 | 43,6 | 34,3 | 42,7 | 640,6 | 694 | 175,4 | 131 | 9,1 | 20 | 1324 | 1391,3 | 2,1 | 2,1 | 45 | 63 |
| **38** | m | 59 | 77,1 | 90,7 | 38,8 | 32,5 | 45,7 | 41,4 | 777,9 | 661,4 | 150 | 214,2 | 23,9 | 18,4 | 794,3 | 411,2 | 1,5 | 1,5 | 63 | 59 |
| **39** | f | 26 | 110,5 | 96,5 | 30 | 44,3 | 27,7 | 35,6 | 542,9 | 621,6 | 298,4 | 145,3 | 2 | 10,7 | 592,8 | 1291,4 | 2,4 | 1,1 | 52 | 75 |
| **40** | m | 35 | 87,5 | 76 | 58,2 | 56,8 | 75,8 | 52,8 | 658,9 | 789,9 | 87,2 | 80,8 | 63,1 | 33,1 | 1926,4 | 2053,9 | 0,9 | 1,5 | 66 | 75 |
| **41** | m | 30 | 94,2 | 81,3 | 29 | 43,2 | 26 | 49,7 | 637 | 738,4 | 298,4 | 143,5 | 2 | 29,1 | 648,1 | 1060,4 | 2,4 | 1,5 | 44 | 55 |
| **Mean** |  | 37,8 | 88,8 | 79,0 | 40,1 | 50,5 | 43,4 | 53,4 | 677,2 | 782,9 | 183,1 | 111,2 | 21,0 | 33,0 | 1119,1 | 1811,2 | 1,7 | 1,2 | 54,4 | 69,4 |
